# Expression of Carbonic Anhydrase III, a Nucleus Pulposus Phenotypic Marker, is Hypoxia-responsive and Confers Protection from Oxidative Stress-induced Cell Death

**DOI:** 10.1038/s41598-018-23196-7

**Published:** 2018-03-20

**Authors:** Elizabeth S. Silagi, Philip Batista, Irving M. Shapiro, Makarand V. Risbud

**Affiliations:** 1 Program in Cell Biology and Regenerative Medicine, Jefferson College of Biomedical Sciences, Thomas Jefferson University, Philadelphia, PA USA; 2 Department of Orthopaedic Surgery, Sidney Kimmel Medical College, Thomas Jefferson University, Philadelphia, PA USA; 3 Department of Surgery, Sidney Kimmel Medical College, Thomas Jefferson University, Philadelphia, PA USA

## Abstract

The integrity of the avascular nucleus pulposus (NP) phenotype plays a crucial role in the maintenance of intervertebral disc health. While advances have been made to define the molecular phenotype of healthy NP cells, the functional relevance of several of these markers remains unknown. In this study, we test the hypothesis that expression of Carbonic Anhydrase III (CAIII), a marker of the notochordal NP, is hypoxia-responsive and functions as a potent antioxidant without a significant contribution to pH homeostasis. NP, but not annulus fibrosus or end-plate cells, robustly expressed CAIII protein in skeletally mature animals. Although CAIII expression was hypoxia-inducible, we did not observe binding of HIF-1α to select hypoxia-responsive-elements on *Car3* promoter using genomic chromatin-immunoprecipitation. Similarly, analysis of discs from NP-specific HIF-1α null mice suggested that CAIII expression was independent of HIF-1α. Noteworthy, silencing CAIII in NP cells had no effect on extracellular acidification rate, CO_2_ oxidation rate, or intracellular pH, but rather sensitized cells to oxidative stress-induced death mediated through caspase-3. Our data clearly suggests that CAIII serves as an important antioxidant critical in protecting NP cells against oxidative stress-induced injury.

## Introduction

The intervertebral disc is a complex joint that comprises an outer fibrocartilaginous annulus fibrosus (AF) of sclerotomal origin, surrounding a gelatinous notochord-derived nucleus pulposus (NP), and cartilaginous endplates on the superior and inferior junctions with the vertebral bodies. Disturbing the integrity of these distinct tissue compartments, especially the avascular NP, results in the development of intervertebral disc degeneration and associated low back and neck pain, the leading cause of years lived with disability in the United States^1^. For this reason, understanding the molecular mechanisms controlling NP cell physiology and pathophysiology is seminal for developing strategies to treat disc degeneration^2–4^.

It is known that the phenotype of NP cells is largely dictated by their unique embryological origin in addition to the hypoxic, acidic, and hyperosmolar niche in which they reside^5–10^. Recent attempts have been made to define the NP cell phenotype using a panoply of ‘markers’: genes, proteins, and metabolic characteristics that are representative and distinguishing of NP cells^9,11–20^. However, the physiological relevance of several of these phenotypic markers to NP cell function is still unknown. Interestingly, CAIII is one such candidate which has been localized in the notochord and developing NP at mRNA level by *in situ* hybridization, resulting in its consideration as an NP marker^21^. However, expression and localization of CAIII protein in embryonic and adult NP tissue was lacking, and its physiological function remained unknown.

Expression of CAIII had been shown in skeletal muscle, fat, and liver cells where it can contribute up to 8–25% of the total soluble protein in these tissues^22–24^. However, it is important to note that CAIII has about 0.3% of the enzymatic activity (ability to interconvert CO_2_/H_2_O to HCO^3−^/H^+^) compared to the highly active cytosolic isoforms CAI/II^25^. This is caused by major kinetic and structural changes of the active site region of the enzyme that create steric-restriction, decreased proton transfer, and inefficient binding of CO_2_^25,26^. In fact, the *in vivo* function of CAIII is still not known; characterization of a global CAIII knockout mouse showed no apparent phenotype in the analysed tissues in which it is abundantly and specifically expressed^27^. Importantly however, some studies have hypothesized that CAIII may act as an oxyradical scavenger to protect intracellular proteins from permanent damage due to oxidative stress^28–31^. This function of CAIII is highly relevant to NP cells which are vulnerable to oxidative stress during degeneration-related annular fissure or disc herniation.

In this study, we confirm that CAIII protein expression is abundant in NP tissues of both embryonic and mature mice. The specificity of the localization in the NP compartment within intervertebral disc qualifies it as one of the most precise markers of NP cells. Furthermore, contrary to the regulation of CAIX and CAXII isoforms, our *in vitro* experiments and analysis of NP specific HIF-1α conditional knockout mice clearly demonstrate that the hypoxia responsive CAIII expression in NP cells is HIF-1α independent. Importantly, our results show that CAIII does not function as a classical carbonic anhydrase in regulating intracellular pH, but rather, functions as a potent antioxidant by sequestering ROS and protecting cells from oxidative stress-induced and caspase-mediated death.

## Results

### CAIII is selectively expressed in the NP compartment of the intervertebral disc

**Figure 1:**
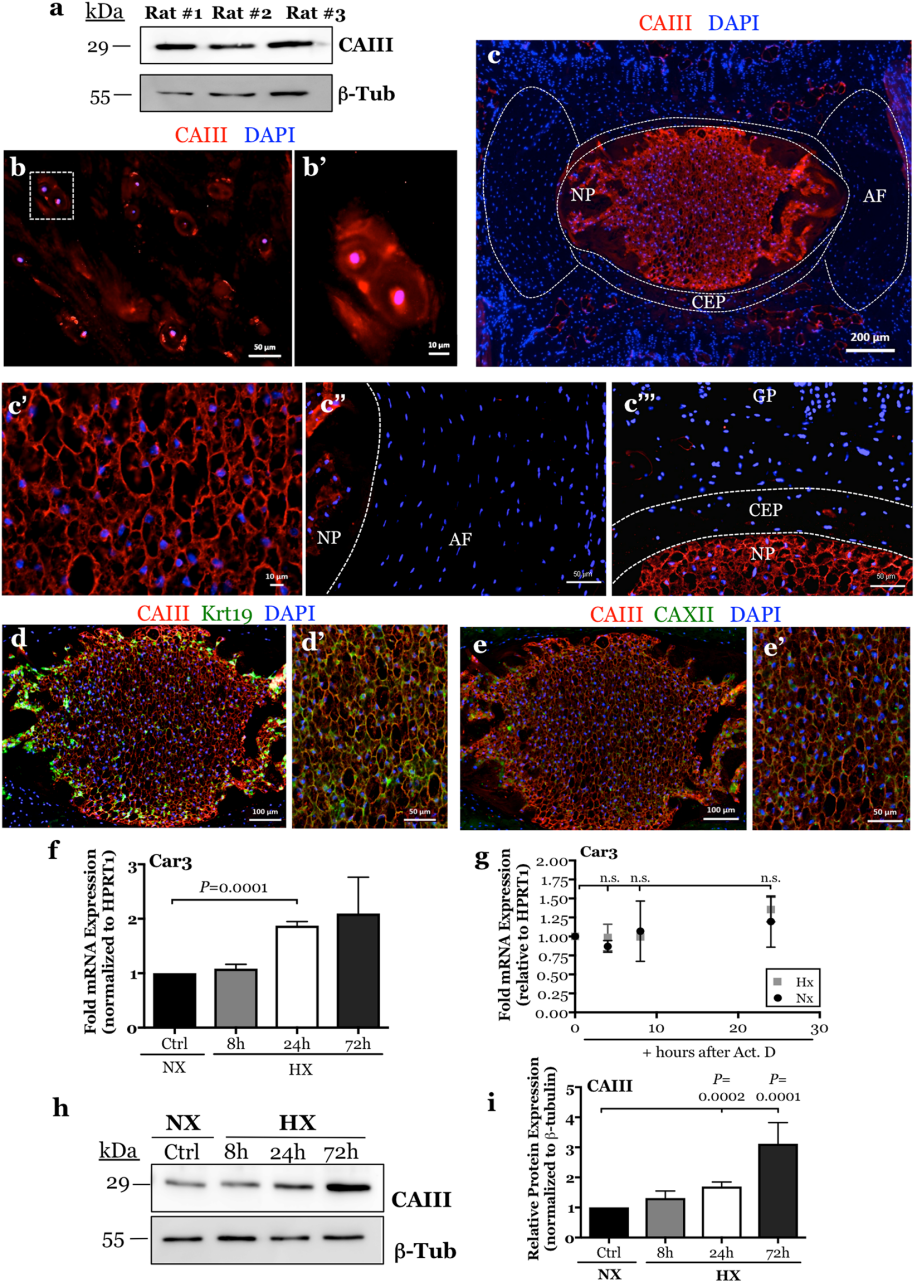
CAIII expression is restricted to NP tissue within the intervertebral disc and is hypoxia-inducible. (**a**) Western blot analysis of CAIII protein shows robust expression in NP tissues from 3 adult rats. (**b**,**b’**) Immunofluorescence detection of CAIII on a transverse section of an adult human intervertebral disc shows specific staining of NP cells. Higher magnification image of cells enclosed by white dotted box shown in (**b’**). (**c**) Immunofluorescence detection of CAIII on a mid-coronal tail section of a 12.5 month-old mouse shows specific staining of the NP tissue. White dotted lines show the distinct NP, AF, and CEP tissue compartments in the IVD. (**c’**-**c”**) Higher magnification images showing CAIII is expressed in the NP (**c’**) and not in the annulus fibrosus (**c”**) or endplate (**c’’’**) compartments of the IVD. (**d**,**e**’) Colocalization of CAIII with known NP cell markers Krt19 (**d**,**d’**) and CAXII (**e**,**e’**) in NP tissue. All cells in the NP compartment were immunopositive for CAIII, Krt19, and CAXII (**d’**,**e’**). (**f**) qRT-PCR analysis of *Car3* mRNA expression in rat NP cells cultured under hypoxia (1% O_2_) for up to 72 hours. (**g**) Actinomycin D chase assay showing *Car3* mRNA stability up to 24 hours after Actinomycin D treatment (5 μg/mL). *Car3* mRNA was highly stable with a half-life (t_1/2_) longer than 24 hours. (**h**) Western blot analysis of CAIII expression in rat NP cells cultured under hypoxia for up to 72 h shows increased protein levels at 24 and 72 hours. (**i**) Densitometric analysis of CAIII from Western blot experiment shown in (**h**). 3 animals were analysed for immunofluorescence. All quantitative data is represented as mean ± SE, n = 4 independent experiments. Oneway ANOVA with Sidak’s multiple comparisons test was used to determine statistical significance. n.s, non-significant. Western blot images were cropped and acquired under same experimental conditions. See Supplementary Fig. S1 for examples of uncropped images for each antibody.

In order to confirm the presence of CAIII in the intervertebral disc, we isolated total protein from the NP tissue of adult rats. Western blot analysis confirmed the robust expression of CAIII protein in the native NP tissue (Fig. 1a). Furthermore, we immunostained transverse sections of a healthy human intervertebral disc with antibodies against CAIII (Fig. 1b,b’). CAIII is strongly expressed by all NP cells, whereas no detectable staining was observed in the annulus fibrosus. To elucidate if expression was conserved across species and to delineate tissue and cellular localization of CAIII, coronal sections of intervertebral discs from 12.5 month-old mice were immunostained with antibodies against CAIII (Fig. 1c-c”). CAIII is robustly and exclusively expressed in the NP tissue compartment with no detectable expression in any of the surrounding tissue compartments including the annulus fibrosus, end-plate and growth-plate (Fig. 1c-c”). Furthermore, staining of NP was homogenous in that each cell was visibly immuno-positive for CAIII (Fig. 1c’). To further confirm its NP-specific localization, we co-immunolabeled the intervertebral disc sections with CAIII and known NP markers Krt19 and CAXII (Fig. 1d-e’). These studies showed that CAIII and Krt19 (Fig. 1d,d’) or CAXII (Fig. 1e,e’) co-labeled all NP cells and demonstrated its utility as a specific NP maker.

### Expression of CAIII is hypoxia-inducible in NP cells

Given the robust expression of CAIII in NP tissue, we investigated the effect of oxygen tension on its mRNA expression and stability. We cultured primary rat NP cells under hypoxic conditions for 8–72 hours and measured mRNA and protein expression by qRT-PCR and Western blot to delineate their dependence on oxygen tension. *Car3* mRNA showed significant hypoxic induction at 24 hours, the trend of increasing expression was also seen at 72 hours (Fig. 1f). However, an Actinomycin D chase assay showed that there was no significant difference in *Car3* mRNA stability under hypoxia or normoxia. Surprisingly, *Car3* mRNA was highly stable such that the half-life (t_1/2_) was more than 24 hours and could not be detected precisely due to toxicity of Actinomycin D (Fig. 1g). It is plausible that the high stability of *Car3* mRNA may be reflective of its robust expression and important biological function in NP cells. Likewise, CAIII protein levels were also elevated at 24 and 72 hours under hypoxic culture (Fig. 1h,i). These results show that CAIII mRNA and protein levels are sensitive to changes in oxygen tension and their expression was up-regulated under hypoxic conditions.

### CAIII expression is insensitive to HIF-1α and HIF-1α does not bind to Car3 promoter

**Figure 2:**
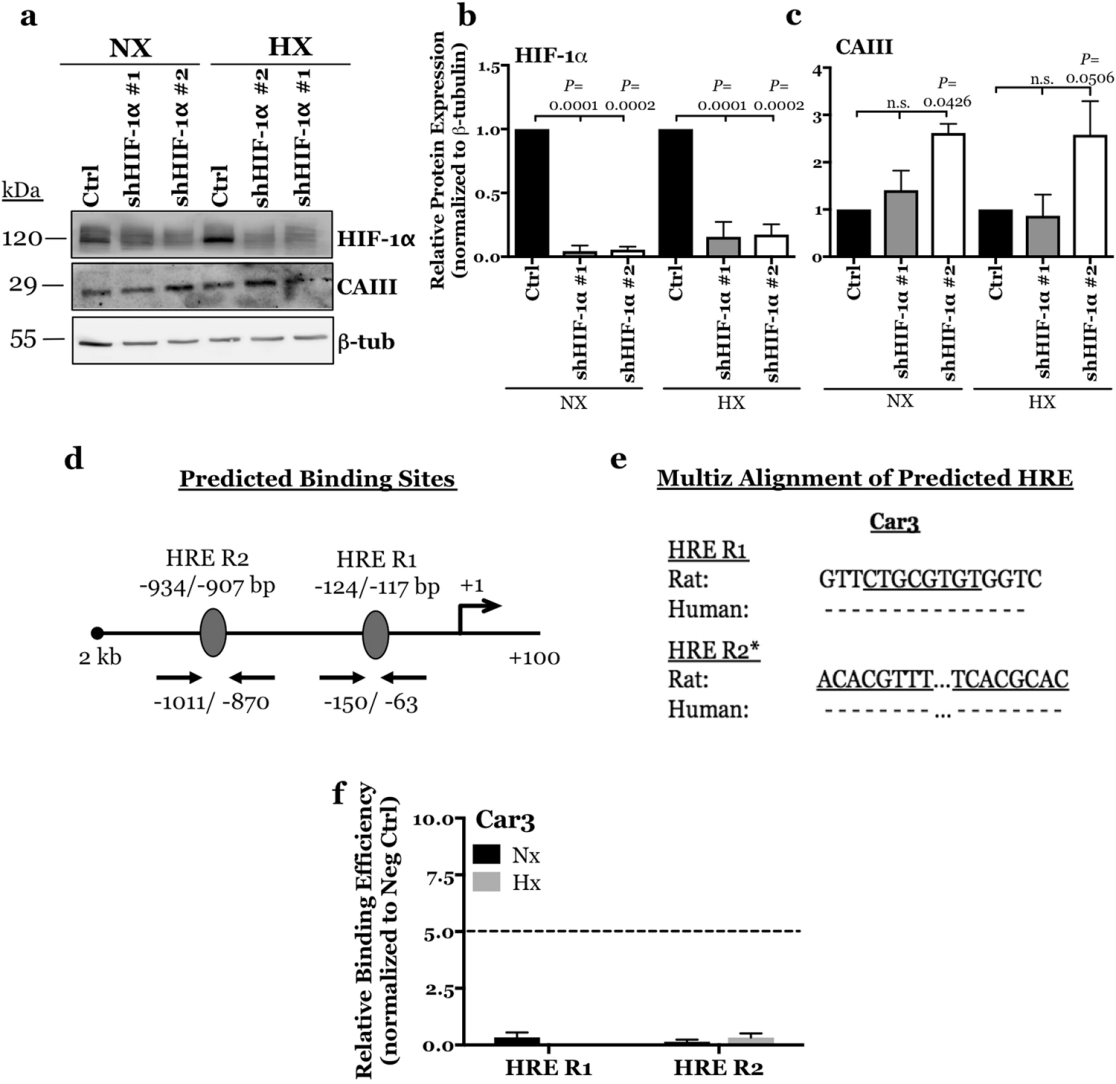
CAIII expression is insensitive to HIF-1α silencing and HIF-1α does not bind to *Car3* promoter. (**a**) Representative Western blot of HIF-1α and CAIII expression in rat NP cells after silencing HIF-1α with lentiviruses expressing independent HIF-1α targeting shRNAs (shHIF-1α #1 and shHIF-1α #2). (**b**,**c**) Densitometric analysis of HIF-1α (**b**) and CAIII (**c**) protein expression from Western blot showed in (**a**). (**d**) JASPAR predicted HRE regions (5′-[A/G]CGTG-3′) within −2000/+100 bp into the rat Car3 promoter with a relative score threshold of 0.85. Predicted HRE regions; ovals. (**e**) Multiz alignment from Ensembl lastz database. Core matrix binding locations are underlined, inability to match shown by (−), *sequences are from the negative strand. (**f**) Relative binding efficiency of HIF-1α to putative HRE regions in the rat *Car3* promoter determined by ChIP. HIF-1α binding to HREs in the rat *Car3* promoter was lacking as enrichment was less than 5-fold than negative control. Data represented as mean ± SE, n ≥ 3 independent experiments. Oneway ANOVA with Sidak’s multiple comparisons test was used to determine statistical significance. n.s, non-significant.

It is known that HIF-1α is a master regulator of many genes important for maintaining NP cell function and phenotype within the hypoxic niche of the intervertebral disc. Importantly, HIF-1α shows stabilized expression in the NP irrespective of oxygen tension^8,9^. Since expression of CAIII in NP cells is hypoxia sensitive, we sought to delineate the relationship between CAIII and HIF-1α using loss of function approaches. We transduced rat NP cells with lentiviruses expressing two HIF-1α shRNA sequences and a control shRNA and measured expression of CAIII following culture under both normoxic and hypoxic conditions. Cells transduced with shHIF-1α showed a ~90% knock-down in HIF-1α levels (Fig. 2a,b). Interestingly, while there was some increase in CAIII protein levels after knock-down with one of the clones, this induction was lacking in cells transduced with the other shHIF-1α clone (Fig. 2a,c). These results were thus inconclusive in determining the relationship between CAIII expression and HIF-1α. It is known that HIF-1α transactivates downstream targets by binding to hypoxia-response elements (HREs) in gene promoters, we therefore used a chromatin immunoprecipitation assay to investigate if HIF-1α binds to the *Car3* promoter. The precise locations of potential HRE consensus sequence (5′-[A/G]CGTG-3′) within rat *Car3* promoter were predicted using JASPAR CORE database, a transcription factor binding prediction tool^32^. Analysis of the first 2 kb (−2000/+100 bp) identified two putative HRE binding sites in the *Car3* promoter (Fig. 2d). When sequence conservation of the putative HREs was analysed using the Multiz alignment tool from Ensembl lastz database (http://www.ensembl.org/index.html), no conservation between rat and human sequences was seen for either HRE R1 or R2 (Fig. 2e) suggesting little physiological relevance of these binding sites. We then investigated whether HIF-1α binds to these predicted HRE regions on *Car3* promoter in rat NP cells using genomic chromatin immunoprecipitation (ChIP). No binding of HIF-1α was detected at either HRE site irrespective of the oxemic tension (Fig. 2f). These results showed that CAIII is not a direct transcriptional target of HIF-1α *in vitro*. In order to gain further insights into *Car3* transcriptional regulation under hypoxia, we analysed 2 kb proximal *Car3* promoter for presence of consensus motifs for transcription factors known to be hypoxia responsive. Bioinformatic analysis showed that *Car3* promoter contained conserved binding sites for ETS1 (−85/−105 bp), NKX3.1 (−401/−419 bp and −1349/−1367 bp), STAT3 (−591/−609 bp), and TWIST1 (−353/−373 bp). It is possible that these transcription factors are responsible for regulating the expression of *Car3* in hypoxic NP cells. Alternatively, a hypoxia-responsive cell type specific enhancer in concert with the promoter may control *Car3* expression in NP cells.

### CAIII expression is unaffected in NP-specific HIF-1α knockout mice

**Figure 3:**
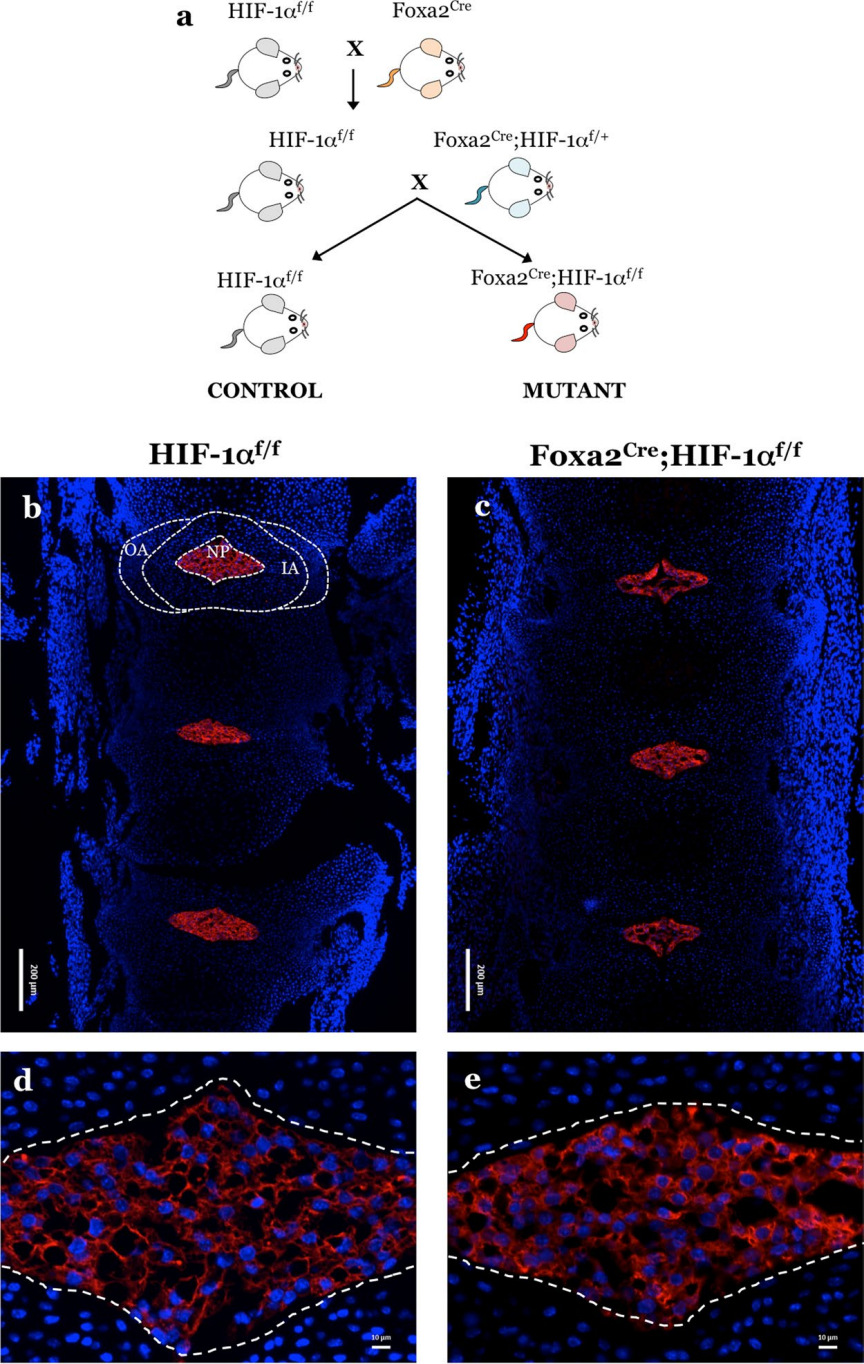
CAIII expression was not affected in mice with NP-specific deletion of HIF-1α. (**a**) Schematic showing generation of NP-specific HIF-1α knockout mice (Foxa2^Cre^; HIF-1α^f/f^) from heterozygous Foxa2^Cre^ and control HIF-1α^f/f^ mice. (**b**-**e**) Representative immunofluorescence images of CAIII expression in E15.5 control (**b**,**d**: HIF-1α^f/f^) and HIF-1α mutant (**c**,**e**: Foxa2^Cre^; HIF-1α^f/f^) littermate mice. Images clearly show NP-restricted localization of CAIII within the intervertebral disc and similar level of expression in control and HIF-1α null mice. Disc compartments denoted by white dashed lines. 3 conditional null and littermate controls were analysed.

To further delineate whether CAIII expression is HIF-1α-dependent *in vivo*, we analysed CAIII expression in NP-specific HIF-1α knockout mice. HIF-1α deletion was achieved by constitutive Cre expression driven by a notochord-specific Foxa2 promoter/enhancer (Fig. 3a)^15^. We chose to analyze CAIII expression at E15.5 since NP cells undergo apoptosis at birth in the HIF-1α conditional null mice^15^. We immunostained disc sections of HIF-1α mutant (Foxa2^Cre^;HIF-1α^f/f^) and control littermates (HIF-1α^f/f^) with CAIII antibody and evaluated the expression by fluorescence microscopy (Fig. 3b–e). We observed that the expression of CAIII in the NP (indicated by the white, dotted line) of HIF-1α mutants (Fig. 3c,e) was unaffected compared to wild-type (HIF-1α^f/f^) (Fig. 3b,d) littermate controls. These results confirmed that CAIII expression was not controlled by HIF-1α signaling in NP. Moreover, staining also corroborated the specificity of CAIII protein as a phenotypic marker of the notochordal NP in the embryonic stage.

### CAIII does not play a role in regulating extracellular acidification rate or intracellular pH

**Figure 4:**
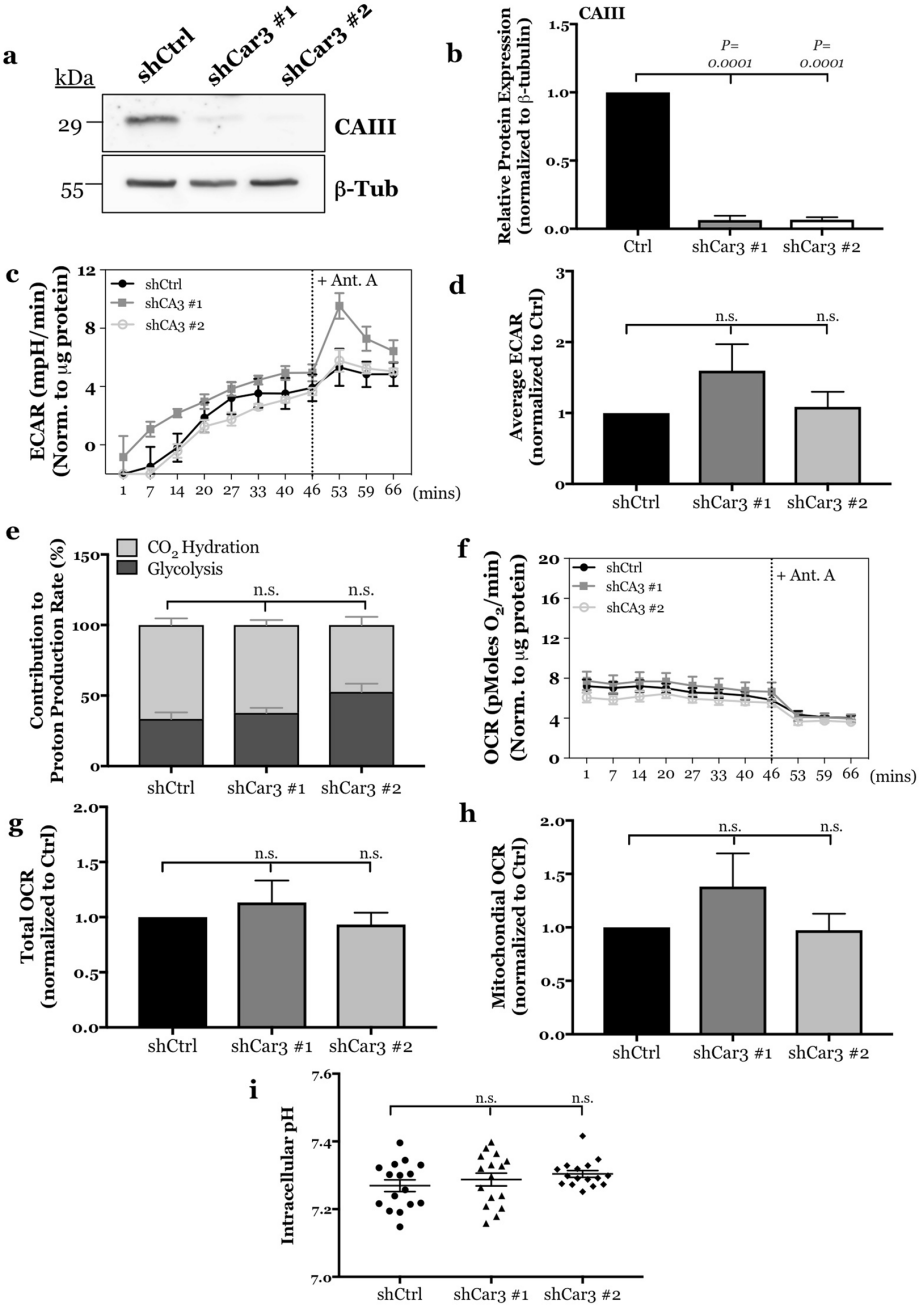
CAIII does not contribute to extracellular acidification rate, CO_2_ hydration rate, or intracellular pH regulation in NP cells. (**a**) Representative Western blot analysis of CAIII protein expression in rat NP cells after silencing CAIII with two independent shRNAs. (**b**) Densitometric analysis of multiple Western blots represented in (**a**). (**c**) Timecourse plot of Seahorse Flux analysis of NP cells showing no effect of CAIII knockdown on ECAR before and after treatment with antimycin A. (**d**) CAIII knockdown did not significantly alter average ECAR before the addition of antimycin A. (**e**) CAIII knockdown had no effect on the relative contribution of glycolysis and CO_2_ hydration to extracellular proton production rate. (**f**) Timecourse plot of Seahorse Flux analysis of NP cells showing no effect of CAIII knockdown on OCR before and after treatment with antimycin A. g, (**h**) CAIII knockdown had no effect on total (**g**) or mitochondrial (**h**) OCR before addition of antimycin A. (**i**) CAIII knockdown had no effect on intracellular pH of rat NP cells. All quantitative data is represented as mean ± SE, n = 3 independent experiments with 4–6 technical replicates per experiment. Oneway ANOVA with Sidak’s multiple comparisons test was used to determine statistical significance. n.s, non-significant.

It has recently been shown that in NP cells, the membrane-associated and extracellularly facing CAIX|XII isoforms are critically important for pH_i_ homeostasis through bicarbonate recycling^33^. In fact, their CO_2_ hydration activity is substantially high such that it contributes up to 66% of the total extracellular proton production rate from NP cells^33^. We investigated whether CAIII functions as a traditional cytosolic carbonic anhydrase despite its known inefficient enzymatic activity^26^. We stably knocked-down CAIII using two independent lentivirally-delivered shRNAs. Western blot analysis confirmed a robust ~90% knock down of CAIII protein in NP cells (Fig. 4a,b). We then measured changes in extracellular acidification rate and oxygen consumption rate with a Seahorse Analyzer in both control and CAIII silenced cells. Interestingly, CAIII silenced cells did not show a decrease in ECAR over time (Fig. 4c,d); treatment with antimycin A had little effect on ECAR, once again underscoring the minimal dependency on electron transport chain in NP cells for ATP generation (Fig. 4c). Moreover, these results demonstrate that silencing CAIII did not alter the contribution of glycolysis or CO_2_ hydration to extracellular proton production rate according to the equation derived by Mookerjee *et al*. and recently applied to NP cells (Fig. 4e)^33,34^. Likewise, silencing CAIII did not affect total OCR (Fig. 4f,g) or mitochondrial OCR (Fig. 4h). Lastly, we measured pH_i_ of rat NP cells after CAIII knock-down using fluorescent pH-sensitive probes. Importantly, the results from this assay showed that pH_i_ of NP cells was not dependent on CAIII activity (Fig. 4i). This result is in contrast to our previous work on membrane-associated extracellularly facing CAIX/XII which showed that pH_i_ in NP cells became more acidic when their activity was inhibited^33^. Taken together, these results clearly suggest that CAIII does not play a role in pH_i_ regulation.

### CAIII protects NP cells from oxidative stress-mediated cell death

**Figure 5:**
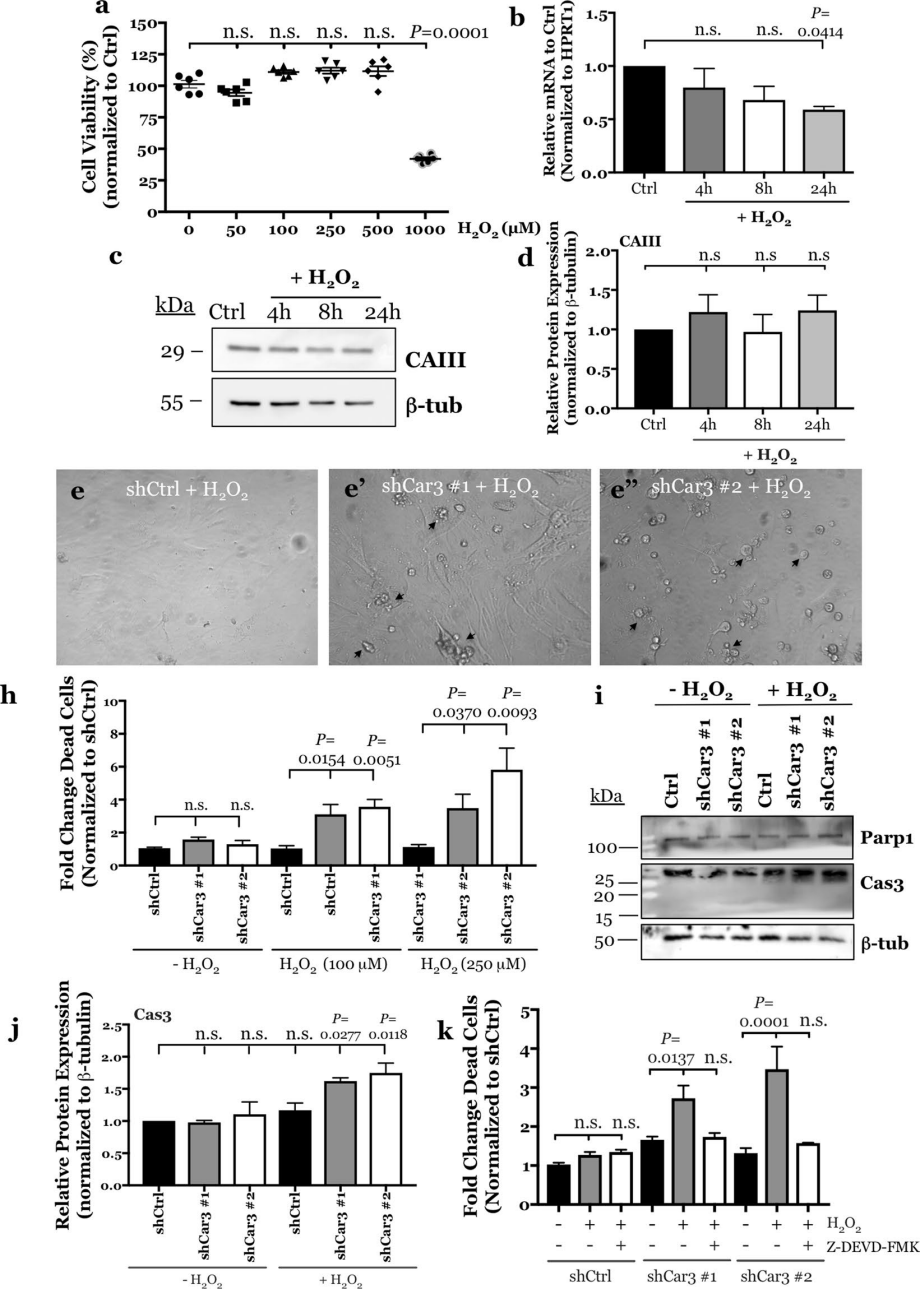
CAIII protects NP cells from oxidative stress-induced, caspase 3-mediated cell death. (**a**) Rat NP cells are resistant to H_2_O_2_-mediated cell death and maintain their viability following treatment with up to 500 μM H_2_O_2_. Viability significantly decreases at 1 mM. (**b**–**d**) Treatment of rat NP cells with 100 μM H_2_O_2_ for 24 hours had no effect on *Car3* mRNA expression analysed by qRT-PCR (**b**) or CAIII protein levels measured by Western blot and subsequent densitometric analyses (**c**,**d**). (**e**) Representative images showing morphology of NP cells transduced with a control shRNA (**e**) and two independent *Car3* shRNAs (**e’**,**e”**) and treated with 100 μM H_2_O_2_ for 24 hours. CAIII silenced cultures showed increased number of dying cells (marked by black arrows). (**h**) CAIII silenced NP cells treated with 100 and 250 μM H_2_O_2_ for 24 hours showed a significant increase in the fold change of dead cells as compared to control populations. (**i**) Representative Western blot images of two markers of apoptosis, total and cleaved Parp1 and Cas3. (**j**) Densitometric analysis of Cas3 protein expression from the Western blot shown in (**i**) shows that total Cas3 expression is significantly increased in CAIII silenced NP cells after treatment with 100 μM H_2_O_2_ for 24 hours. (**k**) Pre-treatment of CAIII silenced NP cells with Cas3 inhibitor, Z-DEVD-FMK blocked H_2_O_2_-induced apoptosis. All quantitative data is represented as mean ± SE, n = 3 independent experiments; 4 technical replicates per experiment were performed when applicable. Oneway ANOVA with Sidak’s multiple comparisons test was used to determine statistical significance. Ordinary twoway ANOVA used to determine statistical significance in Fig. 5k. n.s, non-significant.

There is some evidence in liver and skeletal muscle to suggest that CAIII may function as an oxyradical scavenger of reactive oxygen species (ROS)^28–30^. We therefore investigated whether CAIII plays a similar role as an antioxidant in NP cells. We treated primary rat NP cells with hydrogen peroxide for 24 hours and found that they were surprisingly resistant to death due to hydrogen peroxide (H_2_O_2_)-mediated oxidative stress (Fig. 5a). When exposed to a H_2_O_2_ concentration up to 500 μM cells did not exhibit appreciable decrease in cell viability; cells showed significant decrease in viability at 1 mM (Fig. 5a). However, unlike previous reports in other cell types levels of CAIII mRNA and protein were largely unaffected by 100 μM H_2_O_2_ treatment for 24 hours in NP cells (Fig. 5b–d). To determine whether CAIII, through its oxyradical scavenger activity, protected NP cells from the overt oxidative stress-induced apoptosis, we assessed cell viability in control and CAIII silenced cells after treatment with 100 μM hydrogen peroxide for 24 hours. When compared to shCtrl cells, NP cells transduced with both sh*Car3* clones following H_2_O_2_ treatment exhibited rounded morphology, detachment from substrate, and loss of translucence- telltale signs of dying cells (Fig. 5e-e”). In addition, we measured cell death after 100 and 250 μM H_2_O_2_ treatment in CAIII silenced and control NP cells by quantitatively measuring the fluorescence of DNA-bound Ethidium homodimer-1, which is otherwise impermeable to live cells (Fig. 5h). These results showed that CAIII silenced cells were highly susceptible to cell death in presence of H_2_O_2_ at both concentrations used, supporting findings of the microscopic evaluations. In order to characterize the mechanism of cell death in response to oxidative stress in the CAIII silenced cells, we detected the expression of proteins classically associated with the apoptotic cascade (Fig. 5i). Interesting, total Parp-1 expression was unaffected by 100 μM H_2_O_2_ treatment in CAIII silenced cells, and there was no appreciative accumulation of cleaved Parp-1 (Fig. 5i). On the other hand, treatment of CAIII silenced cells with H_2_O_2_ increased the expression of a total Caspase-3 (Cas3) (Fig. 5i,j). To confirm that the peroxide-induced apoptosis in CAIII silenced cells was mediated by Cas3, we pretreated the cells with a Cas3 inhibitor, Z-DEVD-FMK, before treatment with H_2_O_2_. Indeed, CAIII silenced NP cells pretreated with a Cas3 inhibitor were effectively rescued from H_2_O_2_ -mediated apoptosis (Fig. 5k). Taken together these results strongly suggest that the robust and exclusive expression of CAIII protein in the NP within the intervertebral disc serves to protect NP cells from oxidative stress-induced apoptosis, mediated by Cas3.

## Discussion

CAIII has recently been proposed as a candidate NP phenotypic marker based on its high mRNA expression in notochord and early stages of NP embryogenesis^21^. However, information on the expression and localization of CAIII protein in embryonic and adult NP tissues is lacking. Importantly, the physiological function of CAIII in disc remains elusive. In this study, for the first time, we have demonstrated the validity of CAIII as a faithful NP phenotypic marker and shed light on its role in anti-oxidant defense mechanisms. We have clearly shown the abundant and persistent expression of CAIII in the NP tissue compartment of embryonic and adult animals. When tissue localization of CAIII is compared to that of other known NP markers, CAIII expression is exclusively confined to the notochordal NP and post-natal NP cells within the intervertebral disc, suggesting that it may be important for the maintenance of cell function and phenotype of the NP^33^.

Since the NP niche is defined by low oxygen availability and robust HIF-1 signaling it was logical to explore if hypoxia influenced CAIII expression^35^. Both protein and mRNA expression confirm that CAIII levels were hypoxia-sensitive. Surprisingly, however, *in vitro* loss of function studies were inconclusive in establishing the relationship between HIF-1α activity and CAIII expression. Genomic chromatin immunoprecipitation, however, clearly showed that CAIII was not a direct transcriptional target of HIF-1α as predicted putative HREs failed to bind HIF-1α whereas CAIX, a known target of HIF-1, showed binding under similar conditions^33^. To unequivocally establish the relationship between HIF-1α and CAIII, we analysed the discs of NP-specific HIF-1α conditional null mice. Analysis showed that CAIII protein levels were not affected in null mice thus confirming that CAIII expression in the hypoxic niche of NP is independent of HIF-1α. The insights into plausible factors regulating the hypoxia-sensitive expression of CAIII was forthcoming from analysis of transcription factor binding sites within proximal 2 kb Car3 promoter. This analysis indicated that four hypoxia-sensitive transcription factors, namely EST1^36^, NKX3.1^37^, STAT3^38^, and TWIST1^39^, have conserved binding motifs in the rat as well as human promoters. Based on these findings we hypothesize that the hypoxia-sensitive expression of CAIII in NP cells may either be regulated by these transcription factors together or individually. Alternatively there could be a hypoxia-responsive NP cell-specific enhancer that controls the expression.

In the hypoxic niche of the NP, tight regulation of intracellular pH is critical for maintenance of cell function and viability^33,40–42^. Interestingly, our recent work has shown that NP cells highly express the hypoxia-inducible isoforms CAIX and CAXII in order to regulate pH_i_ through a coordinated bicarbonate recycling metabolon^33^. In fact, the activity of these plasma membrane associated isoforms is sufficiently high such that a majority of extracellular protons are produced by extracellular CO_2_ hydration. While CAIII activity is about 0.3% of other highly active cytosolic isoforms (CAI/II) due to structural differences in the active site^25,26^, it was nonetheless important to determine if it functioned as classical CA in the context of NP. Seahorse flux analysis data of CAIII knock-down NP cells showed little change in oxygen consumption rate, extracellular acidification rate and importantly CO_2_ hydration rate, which should decrease if it was relevant in the production of cytosolic CO_2_ for the bicarbonate recycling metabolon (mediated by HIF-1α, CA9/12, and bicarbonate cotransporters). Furthermore, maintenance of pH_i_ in CAIII silenced cells confirmed that the cytosolic CAIII does not play a role intracellular pH homeostasis.

**Figure 6:**
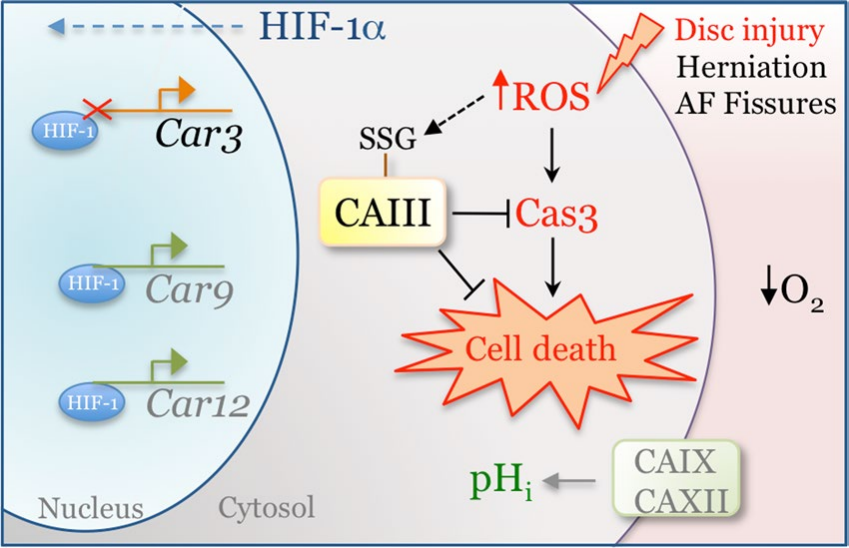
Schematic of the regulation and function of CAIII in NP cells. Hypoxia-responsive CAIII expression in NP cells in independent of HIF-1 activity. S-glutathionylated CAIII primarily functions as an anti-oxidant in protecting cells from oxidative-stress induced apoptosis and does not contribute to intracellular pH homeostasis. This is different from the previously reported HIF-1α-dependent CA isoforms IX and XII which primarily function in intracellular pH regulation.

We therefore asked the question: what is the physiological relevance of CAIII in NP cells? It is well known that the degeneration of intervertebral disc tissues predisposes the disc for annular fissures and ultimately herniation. Disc herniation is associated with a burst of oxygen influx in the disc as well as infiltration and activation of immune cells, both of which may lead to oxidative stress/ROS production in the otherwise hypoxic and immune privileged nucleus pulposus^4,43^. Although it is known that culturing NP cells under 20% O_2_ tension has no conspicuous effect on cell viability, it has been shown that human NP cells cultured under 20% O_2_ tension produced significantly higher levels of mitochondrial-derived ROS as compared to cells in 5% O_2_^44^. This suggests that exposure of NP cells to systemic oxygen levels would cause a significant increase in ROS production in the intervertebral disc. It has also been found that a biomarker of oxidative stress, carboxymethyl-lysine, is accumulated in the proteins of the disc with aging, and that ingestion of pro-oxidative AGEs accelerated degenerative changes in discs^45,46^. In further regard to aging and oxidative stress, treating accelerated aging mice, Ercc1(−/Δ), with a ROS scavenger rescued the matrix catabolism in NP tissue, and glutathione treatment protected NP cells from apoptosis induced by high concentration of H_2_O_2_^44,47^. It is thus important to note that cysteine residues on the surface of the CAIII protein are S-glutathionylated in a process that protects cellular proteins from irreversible damage by oxidative stress^48^. Therefore, the known resilience of NP cells to oxidative stress or H_2_O_2_ treatment clearly suggested that these cells have robust mechanisms to counteract oxidative stress. The contribution of CAIII to NP antioxidant defense was clearly evident from experiments that showed that CAIII silenced cells were highly sensitized to oxidative stress-dependent apoptosis through Cas3 activation. A definitive mechanistic link between CAIII and Cas3 activation was forthcoming from a study that showed protection of CAIII knockout NP cells from apoptosis in presence of Cas3 inhibitor Z-DEVD-FMK. Therefore, it is reasonable to assume that, in the presence of abundant glutathione, the robust levels of CAIII protein in the NP serve as an intrinsic security mechanism protecting the cells from oxidative stress in the event of disc herniation or overt inflammation (Fig. 6). Therefore, maintaining S-glutathionylated CAIII protein levels in NP cells may be a novel therapeutic strategy to combat the effects of oxidative stress associated with aging and degeneration of the intervertebral disc.

## Materials and Methods

### Isolation of NP cells, cell treatments and hypoxic culture

All procedures regarding collection of animal tissues was performed as per approved protocols by Institutional Animal Care and Use Committee (IACUC) of the Thomas Jefferson University, in accordance with the IACUC’s relevant guidelines and regulations. Rat NP cells were isolated as previously reported by our lab^8^. Cells were maintained in Dulbecco’s Modification of Eagle’s Medium (DMEM) supplemented with 10% FBS and antibiotics. Cells were cultured in a Hypoxia Work Station (Invivo2 300, Ruskinn, UK) with a mixture of 1% O_2_, 5% CO_2_ and 94% N_2_. To investigate the effect of oxidative stress, NP cells were treated with hydrogen peroxide (H_2_O_2_) (250 μM) for 24 hours. To assess the role of Cas3 in peroxide-induced apoptosis, we treated NP cells with Cas3 inhibitor (Z-DEVD-FMK) (50 μM) for 2 hours before treatment with H_2_O_2_ for 24 hours.

### Protein extraction, Immunoprecipitation, and Western Blotting

Following treatment, cells were washed on ice with ice-cold 1× PBS with protease inhibitor cocktail (Thermo Scientific). Cell were lysed with lysis buffer containing 1× protease inhibitor cocktail (Thermo Scientific), NaF (4 mM), Na_3_VO_4_ (20 mM), NaCl (150 mM), β-glycerophosphate (50 mM), and DTT (0.2 mM). Total protein was resolved on 10% SDS-polyacrylamide gels and transferred to PVDF membranes (Fisher Scientific). Membranes were blocked with 5% nonfat dry milk in TBST (50 mM Tris pH 7.6, 150 mM NaCl, 0.1% Tween 20) and incubated overnight at 4 °C in 5% nonfat dry milk in TBST with anti-HIF-1α (1:500, R&D Systems); anti-CAIII (1:500, SCBT), anti-Parp1 (1:1000, Cell Signaling), anti-Cas3 (1:1000, Cell Signaling), or anti-β-tubulin (1:5000, DSHB) antibodies. Specificity of all antibodies has been validated by the manufacturers using siRNA or negative control IgG. Immunolabeling was detected using ECL reagent (LAS4000, GE Life Sciences). Densitometric analysis was performed using ImageQuant TL (GE Life Sciences). All quantitative data is represented as mean ± SE, n ≥ 4 independent experiments.

### Immunohistological analysis

Healthy human L3-L4 disc from 33-year old female was obtained from the Cooperative Human Tissue Network (CHTN), an NCI supported resource. 12.5-month old mouse tails from a mixed Swiss/129SvEv genetic background were harvested and fixed in 4% PFA for 24 hours and decalcified in 12.5% EDTA at 4 °C for 6 weeks prior to paraffin embedding. Coronal sections, 7 μm in thickness, were deparaffinized, rehydrated in graded alcohols, and an antigen retrieval step was performed with heated citrate buffer (pH 6) for 20 minutes. Slides were blocked in 10% fetal bovine serum in PBST (1× PBS, 0.4% Triton-X) for 1 h at room temperature. Sections were then sequentially incubated with antibodies CAIII (1:100, SCBT) and CAXII (1:500, Cell Signaling) or Keratin 19 (1:3, DHSB Hybridoma Product TROMA-III), a in 10% fetal bovine serum in PBST at 4 °C overnight^11^. After thoroughly washing, the sections were incubated with Alexa Fluor-594 AffiniPure F(ab’)2 conjugated anti-goat, anti-rabbit or anti-rat secondary antibodies (Jackson ImmunoResearch) for 1 h at room temperature. Sections were visualized using a fluorescence microscope (AxioImager A2, Zeiss) fitted with a monochromatic camera (AxioCam MRm, Zeiss). n = 3 independent experiments were analysed for each antibody.

### Real Time RT-PCR Analysis

Total RNA was extracted from NP cells using RNAeasy mini columns (Qiagen). Purified DNA-free RNA was converted to cDNA using EcoDry™ Premix (Clontech). Equal amounts of template cDNA and gene-specific primers were incorporated into a SYBR Green master mixture (Applied Biosystems) and mRNA expression was quantified using the Step One Plus Real-time PCR System (Applied Biosystems). HPRT was used to normalize gene expression. Melting curves were analysed to verify the specificity of the RT-PCR and the absence of primer dimer formation. Each sample was d in duplicate and included a template- free control. All primers used were synthesized by Integrated DNA Technologies, Inc. All quantitative data is represented as mean ± SE, n ≥ 4 independent experiments.

### Actinomycin D Chase

Rat NP cells were treated with Actinomycin D (5 μg/mL) for 0, 4, 8 and 24 hours. Total RNA was extracted from cells as described in the Real Time RT-PCR Analysis method section. Data is represented as mean ± SE, n = 4 independent experiments.

### Plasmids and Reagents

LV-shHIF-1α (#54450 designated clone 1, #232222 designated clone 2) and control pLKO.1 were purchased from Sigma-Aldrich. *Car3* pGFP-C-shLenti (unique 29mer shRNA constructs in lentiviral GFP vector; clone A designated clone #1, clone C designated clone #2) and shCtrl (non-effective 29-mer scrambled shRNA cassette in pGFP-C-shLenti vector) were purchased from Origene (Rockville, MD). The following plasmids were obtained from the Addgene repository: psPAX2 (catalogue no. 12260) and pMD2G (catalogue no. 12259) developed by Dr. Didier Trono.

### Bioinformatics Analysis

The nucleotide sequence of the 2 kb proximal promoter of rat *Car3* gene was found using the UCSC Table Browser^49^. Putative HRE consensus sequences (5′-[A/G]CGTG-3′) were determined using the JASPAR Core Database (http://jaspar.genereg.net/) with a relative score threshold of 0.85^32^. Multiz alignment of HRE motifs was performed using the Ensembl Lastz Database to analyse species conservation (http://www.ensembl.org/index.html). In addition, we performed bioinformatic analysis of transcription factor binding sites on the 2 kb proximal promoter of Car3 using MatInspector (Genomatix Software Suite) with a matrix-similarity threshold of 0.85 and TF family p-value of ~0.05 or below.

### Chromatin Immunoprecipitation

Rat NP cells were plated in 15-cm plates and cultured under normoxic or hypoxic conditions for 24 hours. ChIP assay was performed using ChIP-IT® high sensitivity kit (Active Motif, Carlsbad, CA) according to the manufacturer’s recommendations. Cells were lysed and chromatin sheared by sonication. Input DNA was generated by treating aliquots with RNase, proteinase K, and heat, followed by ethanol precipitation. DNA complexes were immunoprecipitated by incubation with anti-HIF-1α antibody (Cell Signaling) overnight at 4 °C followed by binding to protein G-agarose beads for 3 h at 4 °C. Cross-links were reversed by treatment with proteinase K and heat for 2.5 h, and DNA was purified using DNA purification elution buffer. Real time PCR analysis was performed using ChIP-IT® quantitative PCR analysis kit (Active Motif) using the primer pairs for putative HRE sites as shown in Supplementary Table S1. Negative control primers and standard curve primers used were provided with kit. Real time PCR was performed with Power SYBR® Green PCR Master Mix (Applied Biosystems). The *C*_t_ values were recorded, and the data were normalized based on primer efficiency, input DNA *C*_t_ values, amount of chromatin, and re-suspension volume, based on manufacturer’s recommendations. Criteria for positive binding include ≥5 HIF-1α binding events/1,000 cells and binding efficiency 5-fold greater than negative control. Data is represented as mean ± SE, n ≥ 3 independent experiments.

### Lentiviral Particle Production and Viral Transduction

HEK293T cells were seeded in 15-cm plates (3 × 10^6^ cells/plate) in Opti-MEM (Life Technologies) with 2% FBS 2 days before transfection. The cells were transfected with 18 μg of control shRNA (pLKO.1 or pGFP-C-shLenti shCtrl) and shRNA against HIF-1α (shHIF-1α) or *Car3* (sh*Car3*) plasmids along with 12 μg of psPAX2 and 6 μg of pMD2.G. After 16 h, the transfection medium was removed and replaced with DMEM with 10% heat-inactivated FBS and penicillin-streptomycin. Lentiviral particles were harvested at 48 h and 60 h post-transfection. NP cells were plated in DMEM with 10% heat-inactivated FBS 1 day before transduction. Cells in 10-cm plates were transduced with 5 ml of conditioned media containing viral particles along with 8 μg/ml Polybrene. After 24–48 h, media was removed and replaced with DMEM with 10% FBS and continued for 3 days. The cells were cultured in hypoxia or normoxia for additional 24 h and harvested for protein and mRNA extraction 5 days after transduction.

### Seahorse XF Analyzer Respiratory Assay

The Seahorse XF24 instrument was used to measure extracellular acidification rate (ECAR) and O_2_ consumption rate (OCR), as reported by Csordás *et al*.^50^. Rat NP cells transduced with either shCtrl or sh*Car3* plasmids were seeded on 24-well XF Analyzer plate at 15,000 cells per well in DMEM (5 mM glucose, 4 mM glutamine, pH 7.4 @ 37 C). The microplate was incubated for 1 h in no CO_2_ at 37 °C. OCR was calculated using the Akos algorithm, a standard algorithm which we determined was appropriate even for the low OCR readings that we recorded in NP cells; appropriateness was based on the approximate linearity of the pO_2_ vs time traces. ECAR was measured from readings of H^+^ concentration. After each measurement, the probe array rises, after which the solution in each well was mixed for 2 min (by gently moving the probe array up and down) to remove O_2_ and metabolite gradients, followed by a 2-min waiting period before the next measurement phase (i.e., lowering of the probe). Our experiments included 8 OCR and ECAR measurements to create a baseline, followed by the injection of Antimycin A (Sigma Aldrich). 3 OCR and ECAR measurements were then made. All measurements were normalized to total protein concentration using a standard BCA assay. Mitochondrial OCR was calculated by subtracting the final OCR value (pMoles O_2_/min) after Antimycin A treatment from the average of the 3 OCR values before Antimycin A treatment. All quantitative data is represented as mean ± SE, n = 3 independent experiments; 4–6 technical replicates per experiment.

### Intracellular pH Measurement

Rat NP cells transduced with either shCtrl or sh*Car3* plasmids were plated in a 96-well plate at 5,000 cells per well. Intracellular pH was measured following the pHrodo Red AM Intracellular pH Indicator (ThermoFisher) protocol. A standard curve was calculated by clamping the intracellular pH with buffers at a pH of 4.5, 5.5, 6.5, and 7.5 after treatment with a 10 μM valinomycin/nigericin ionophore cocktail. All quantitative data is represented as mean ± SE, n = 3 independent experiments; 4 technical replicates per experiment.

### Cell Death Assay

Rat NP cells transduced with either shCtrl or sh*Car3* plasmids were plated in a 96-well plate at 5,000 cells per well. Cells were treated with H_2_O_2_ for 24 hours with or without pretreatment with Caspase-3 inhibitor. Viability was measured by Calcein-AM and Ethidium homodimer −1 dye. Fold change in dead cells was measured using Ethidium homodimer −1 dye following standard protocol. All quantitative data is represented as mean ± SE, n = 3 independent experiments; 4 technical replicates per experiment.

### Statistical analysis

All experiments were performed in triplicate at minimum, and data are presented as mean ± S.E. Differences between groups were analysed by the one-way and two-way ANOVA depending on the number of variables with appropriate post-hoc analyses (Sidak’s and Tukey’s multiple comparisons test) using Prism7 (Graphpad Software); p < 0.05.

### Data Availability

All data generated or analysed during this study are included in this published article (and its Supplementary Information files).

## Electronic supplementary material

Supplementary Information
