# Supplementary material for: Expression of Carbonic Anhydrase III, a Nucleus Pulposus Phenotypic Marker, is Hypoxia-responsive and Confers Protection from Oxidative Stress-induced Cell Death
Source: Sci Rep. 2018 Mar 20;8:4856. doi: 10.1038/s41598-018-23196-7 (PMC5861082; doi:10.1038/s41598-018-23196-7)
Supplement: Supplementary file 1 — Supplementary Information [file 41598_2018_23196_MOESM1_ESM.pdf]

Supplementary Information

Expression of Carbonic Anhydrase III, a Nucleus Pulposus Phenotypic Marker, is Hypoxia-responsive and Confers Protection from Oxidative Stress-induced Cell Death

Elizabeth S. Silagi[1], Philip Batista[2], Irving M. Shapiro[1,2], Makarand V. Risbud[1,2]

Supplementary Figure S1-1. Examples of Uncropped Western Blots

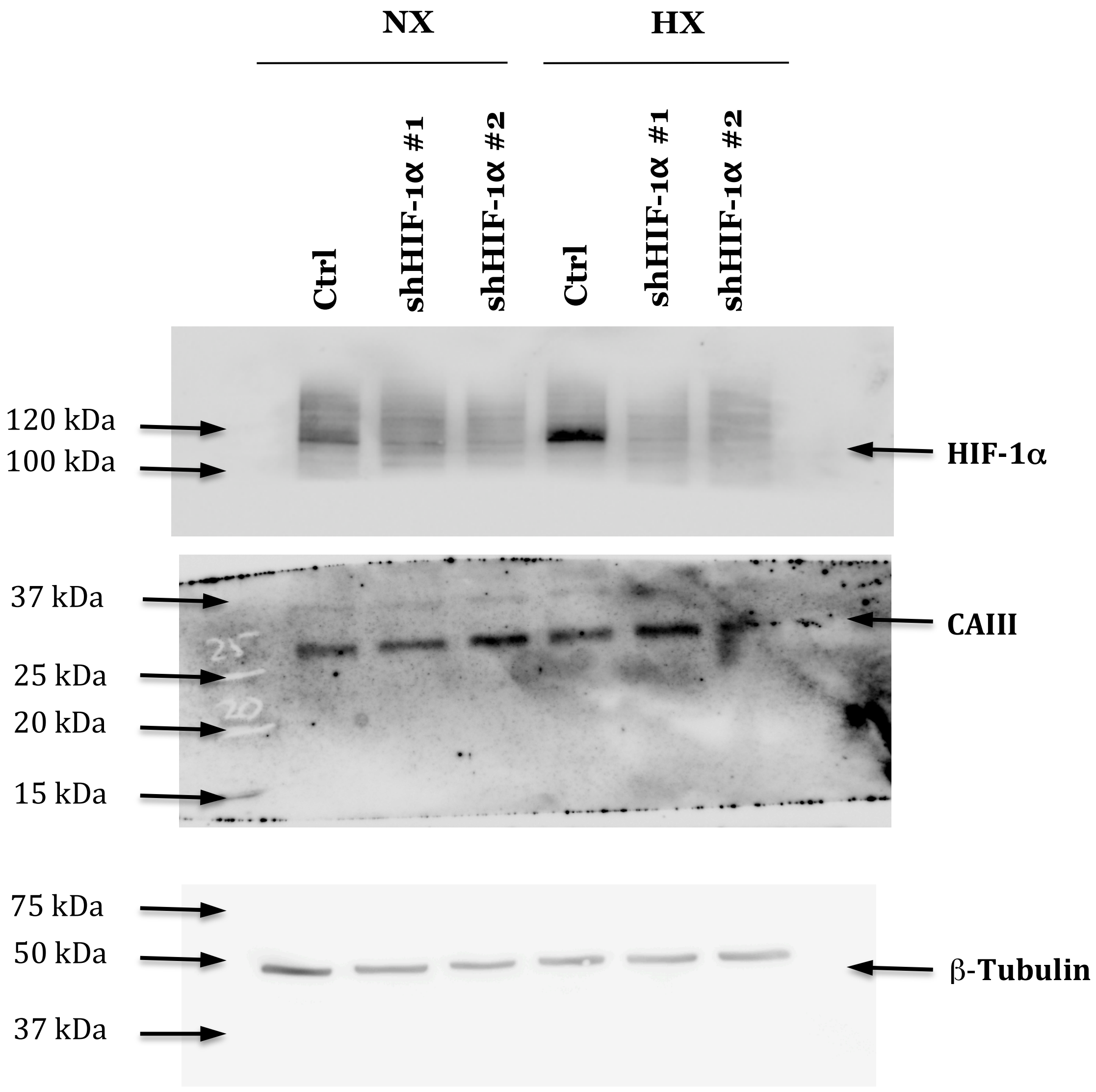

Examples of Uncropped Western Blots for HIF-1α, CAIII, and β-Tubulin from Figure 2a.

Supplementary Figure S1-2. Examples of Uncropped Western Blots

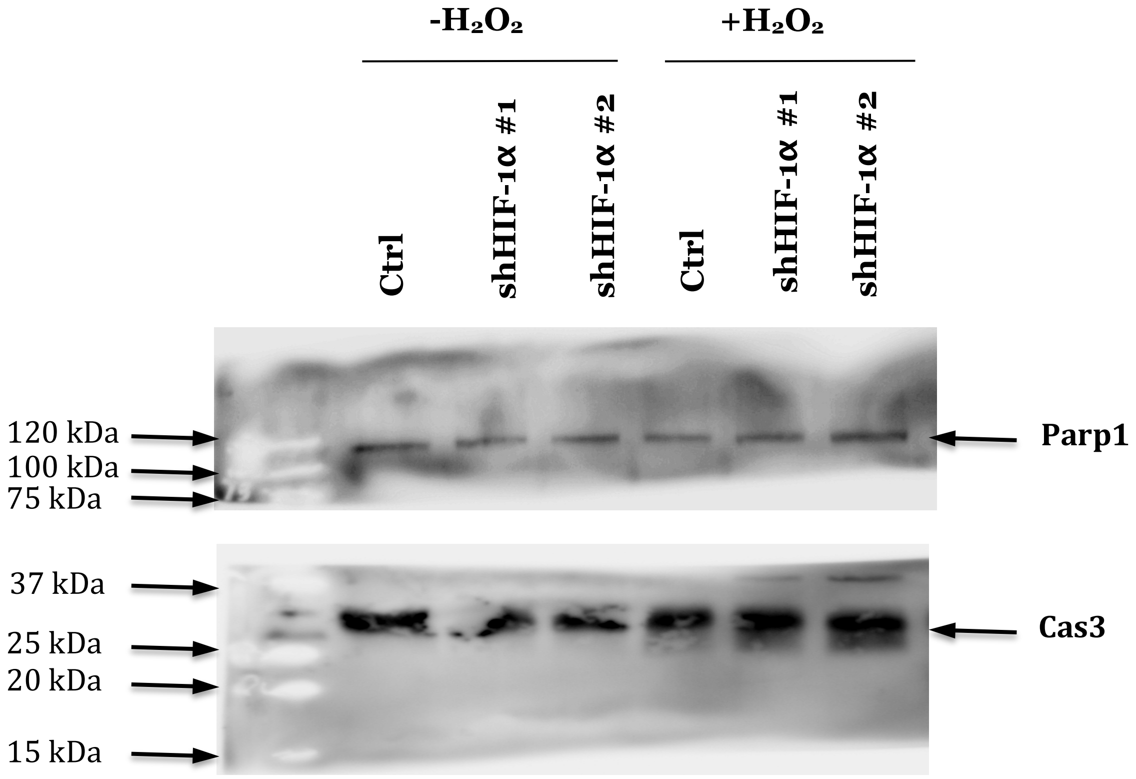

Examples of Uncropped Western Blots for Parp1 and Cas3 from Figure 5i.

Supplementary Table S1: Primer sequences used for Chromatin Immunoprecipitations

Gene Name	Primer Location		Primer Sequence	
**HRE Region**	**Start**	**End**	**Forward**	**Reverse**
Car3 R1	-63	-150	5′-agtgatccctggtcacaaaa-3′	5′-cggctaagtatgtggaatgg-3′
Car3 R2	-870	-1011	5′-gtcccagattcctcctcatc-3′	5′-cagtctctggacgcttgagt-3′
